# Small Molecules as Protein-Protein Interaction Inhibitors

**Published:** 2016

**Authors:** Zahra Hajimahdi

**Affiliations:** *Department of Medicinal Chemistry, School of Pharmacy, Shahid Beheshti University of Medical Sciences, Tehran, Iran*


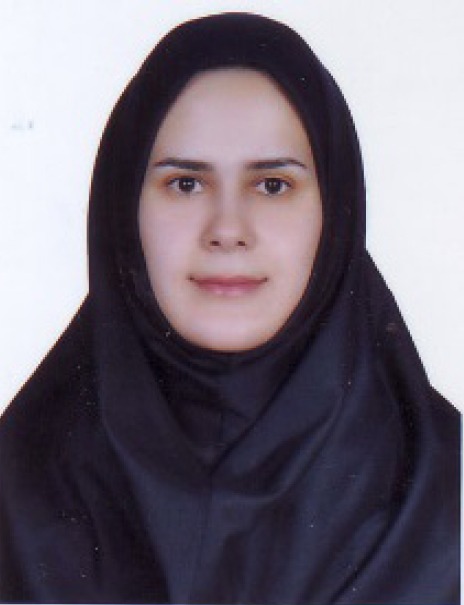


Protein-protein interactions (PPIs) can be considered as a vast class of therapeutic targets. PPIs play critical roles in all biological functions and are often dysregulated in disease. Despite their functional role both inside and outside the cell, over the past decades PPIs have been neglected as undruggable targets. This was in part due to the fact that PPI interfaces are generally flat and large (roughly 1,000–2,000 Å^2^ per side), in contrast to the cavities that typically accommodate small molecules (around 300–500 Å^2^). Another challenge is that unlike enzymes or G protein-coupled receptors there is no endogenous ligands or substrates to start the drug design process. Starting around the years 2000 and up to now, a clinically approved integrin antagonist (tirofiban) and other numerous investigations suggest a hopeful emerging view: small drug-like molecules can modulate PPIs. Mutational analysis of protein interfaces revealed that just some amino acids present at the interfaces were directly responsible for the protein-protein complex stabilization which are defined as hotspot residues. Hotspots tend to be located as clusters around the center of the interface on both protein partners that is often called hot region. Small molecules that interact with these hotspot residues can inhibit protein-protein interactions which kwon as orthosteric inhibitions. 

Experimental HTS (high throughput screening), fragment-based lead discovery and other related biophysical approaches have been used to assist the rational design of low-molecular-weight and low-complexity molecules as PPI inhibitors. 

PPI inhibitors bind at the hotspot and mimic the types of binding interactions made by the partner. Small molecules that are optimal to block PPIs would need to have a 3D structure and shape that allow the distribution of functional groups in several small subpockets. Hence, orthosteric inhibitors are usually larger than 500 Da, more lipophilic, more aromatic and more complex 3D than the average compounds in an HTS library. 

**Figure 1:**
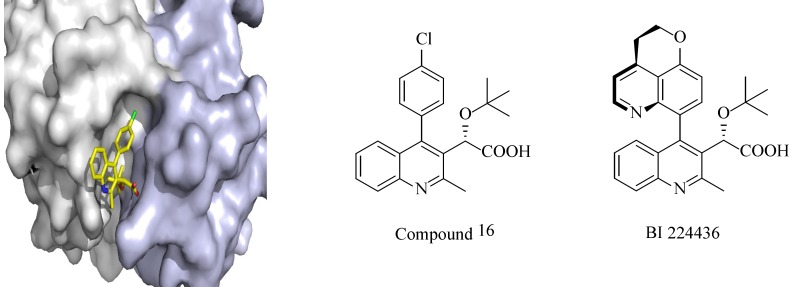
Representation of compound 16 bound to the catalytic core domain of HIV integrase

Over the last decade, about 50 PPIs have been targeted and several inhibitors have been identified and reached clinical trials. One of the examples of PPIs is HIV integrase interaction with LEDGF/p75. Integrase is one of the key enzymes in HIV replication, which mediates integration of the viral cDNA into the host genome by catalytic reactions. Due to its limited genome, HIV needs to use cellular co-factors for efficient replication in the host cell. LEDGF/p75 (The human protein lens endothelial growth factor), a transcriptional co-activator interacts with integrase and facilitates insertion of viral cDNA into the host cell genome, protects integrase from degradation and stimulates its catalytic activity. The cocrystal structure of LEDGF/p75 binding domain with a dimer of integrase catalytic core domain shows the critical residues from both protein partners (L368, I365, D366 and K364 from LEDGF/p75 and A128, H171 and E170 from HIV-IN) with 400 Å^2^ surface area on IN. Different approaches including HTS of diverse chemical libraries, computational database screening of virtual small molecule libraries and structure-based *de novo* design have been employed to design and identify small-molecule inhibitors of the LEDGF/p75–IN interaction. Albeit different classes of LEDGF/p75–IN interaction inhibitors have been described so far, the most potent inhibitors are* tert*-butoxy-(4-phenyl-quinolin-3-yl)-acetic acid (tBPQA) derivatives including the clinical compound BI-224436 which has nanomolar antiviral potency with EC_50_ = 11-27 nM (Figure 1). tBPQA derivatives not only block LEDGF/p75–IN interaction, but also allosterically inhibit IN catalytic activity by preventing the formation of functional IN tetramer. The divergent resistance pathway of these inhibitors in comparison with active site integrase inhibitors and their multimodal mode of actions make them candidates for future HIV-drug discovery.

In summary, recent advances in proteomics, computational chemistry, and ligand design have made PPIs from undruggable to attractive targets for the development of therapeutics. Today, nearly a dozen PPIs inhibitors have been in the clinic. Overall, the future of PPI seems bright and much progress in this area has generated the courage to move forward, even with enthusiasm.

*Zahra Hajimahdi is currently working as an assistant professor at the Department of Medicinal chemistry, School of Pharmacy, Shahid Beheshti University of Medical Sciences, Tehran, Iran. She could be reached at the following e-mail address:* z.hajimahdi@sbmu.ac.ir

